# α-Fe_2_O_3_ Nanoparticles Aided-Dual Conversion for Self-Powered Bio-Based Photodetector

**DOI:** 10.3390/nano12071147

**Published:** 2022-03-30

**Authors:** Ishita Chakraborty, Sz-Nian Lai, Jyh-Ming Wu, Chao-Sung Lai

**Affiliations:** 1 Department of Electronic Engineering, Chang Gung University, Guishan District, Taoyuan City 33302, Taiwan; d0627107@cgu.edu.tw; 2 Department of Materials Science and Engineering, National Tsing Hua University, Hsinchu 30010, Taiwan; snlai712@gapp.nthu.edu.tw (S.-N.L.); jmwuyun@gapp.nthu.edu.tw (J.-M.W.); 3 Ph.D. Program in Prospective Functional Materials Industry, National Tsing Hua University, Hsinchu 30010, Taiwan; 4 High Entropy Materials Center, National Tsing Hua University, Hsinchu 30010, Taiwan; 5 Artificial Intelligence and Green Technology Research Center, Chang Gung University, Guishan District, Taoyuan City 33302, Taiwan; 6 Department of Materials Engineering, Ming Chi University of Technology, Taishan District, New Taipei City 24301, Taiwan; 7 Department of Nephrology, Chang Gung Memorial Hospital, Guishan District, Taoyuan City 33305, Taiwan

**Keywords:** triboelectric nanogenerator, α-Fe_2_O_3_ nanoparticles, photoelectrons, self-powered photodetector, human hair, circular bio-economy

## Abstract

Eco-friendly energy harvesting from the surrounding environment has been triggered extensive researching enthusiasm due to the threat of global energy crisis and environmental pollutions. By the conversion of mechanical energy that is omnipresent in our environment into electrical energy, triboelectric nanogenerator (TENG) can potentially power up small electronic devices, serves as a self-powered detectors and predominantly, it can minimize the energy crisis by credibly saving the traditional non-renewable energy. In this study, we present a novel bio-based TENG comprising PDMS/α-Fe_2_O_3_ nanocomposite film and a processed human hair-based film, that harvests the vibrating energy and solar energy simultaneously by the integration of triboelectric technology and photoelectric conversion techniques. Upon illumination, the output voltage and current signals rapidly increased by 1.4 times approximately, compared to the dark state. Experimental results reveal that the photo-induced enhancement appears due to the effective charge separation depending on the photosensitivity of the hematite nanoparticles (α-Fe_2_O_3_ nanoparticles) over the near ultraviolet (UV), visible and near infrared (IR) regions. Our work provides a new approach towards the self-powered photo-detection, while developing a propitious green energy resource for the circular bio-economy.

## 1. Introduction

The power generation by harvesting mechanical [1], light [2], thermal [3] and magnetic [4] energy from ambient sources has attracted significant attention because of the advanced development of self-powered techniques, small portable electronics, and wireless sensing networks. Developed on the coupling of contact electrification and electrostatic induction, a triboelectric nanogenerator (TENG) can satisfactorily convert mechanical energy, in particular the energy in vibrations, wind, waves, and frictions produced by regular human motions, into electricity. In principle, any two dissimilar materials with evidently opposite triboelectric polarity are potentially implemented as the tribo-materials in TENG [5]. Because of the superior output power density, superior energy conversion efficiency, lower cost, simple device fabrication, and mechanically robustness numerous potential applications like, environmental surveillance, self-powered sensors (i.e., motion, pressure, and acoustic sensors), power sources for portable and wearable electronic devices have been successfully exhibited [6,7,8,9,10,11,12,13,14].

Although the basic mechanism behind the triboelectric charging phenomena has been considered to be well understood [15], the expeditious development of nanotechnologies along with the vast potential applications has notably increased the requirement to upgrade our apprehension of the mechanism under various realistic conditions [16]. Photo detection is of utmost importance because of various scientific applications like fiber optical communication systems, environmental monitoring, safety and for defense-related issues. On the other hand, the menace of global environmental pollution caused by the traditional fossil fuels and the continuous thrive of global demand for energy, the demand for the eco-friendly and self-powered photo detection over the conventional modules of photo detection is attracting world-wide attention [17]. In this aspect, the integration of triboelectric technology and photoelectric conversion mechanisms can be considered as an effective approach towards the execution of self-powered photo detectors with a simple, cost effective and maintenance free self-subsisting device structure. More importantly, it has been illustrated that both the photoconductivity and surface charge density of some semiconductor materials can be altered because of the superior photoelectric properties of those semiconductor materials by absorbing the solar energy [18]. Polymers are widely preferable materials as the primary dielectric for the fabrication of the self-powered and flexible wearable TENG devices, among them polydimethylsiloxane (PDMS) is appraised to be one of the best choices, owing to its high electronegativity, transparency, inherent elasticity, mixing ability with various nanostructured materials to prepare different composites, its capability of coating other surfaces, its hydrophobic surface and most importantly excellent biocompatibility [19,20]. Hematite nanoparticles (α-Fe_2_O_3_ nanoparticles) are acknowledged as one of the widely applied photocatalysts due to their high activity, light stability, ready availability, low cost, environmental friendliness and corrosion resistance [20,21,22]. After the illumination of UV-visible light, free electrons are excited to the conduction band (CB), thereby leaving holes in the valence band (VB). Electron excitation by photon absorption will influence the possibility of the electron transfer in contact state, thus, the output performance of TENGs. This conception can be appraised for the self-powered photo-detection. On the other hand, several designs of triboelectric energy harvesters relying on recycling natural materials which are abundant in nature have been developed in recent years as eco-friendly solutions to the energy crisis [20,23,24]. Utilizing various materials easily available around us, recognized as waste material in most parts of the world, we can build eco-friendly, biocompatible, and highly efficient devices.

In this study, we describe the hybridization between triboelectricity and photoelectric properties for simultaneous harvesting of the mechanical energy and solar energy based on a flexible and transparent TENG consist of PDMS/α-Fe_2_O_3_ nanocomposite film (as the negative tribo-layer) and processed hair film (as the positive tribo-layer). Human hair, which is well known to be a powerful triboelectric material, are regarded as a slowly degrading waste material and its aggregation in waste streams causes environmental pollution. Thus the use of human hair waste eases a defiance to waste management systems and promotes the development of a propitious green energy source for the circular bio-economy. The as-fabricated bio-TENG exhibits excellent responsivity, rapid response time, appreciable repeatability, and broadband detection ability that extends over the near ultraviolet (UV), visible and near infrared (IR) regions. This work demonstrates a new way to developing high performance self-powered photodetectors, while developing waste hair utilization, that will play a significant part in circumventing climate emergencies.

## 2. Materials and Methods

### 2.1. Hydrothermal Synthesis of α-Fe_2_O_3_ Nanoparticles

First, 156 mg of FeCl_3_·6H_2_O (98.0–102%, Sigma Aldrich, St. Louis, MO, USA) was dissolve in 40 mL of DI water through sonication for around 15 min to prepare a homogenous solution. Afterwards, 5.6 mL of 25% ammonia solution (Honeywell Fluka, Seelze, Germany) was added steadily (dropwise) up to 30 min under constant stirring. Right away, the prepared solution was moved into a Teflon-lined steel autoclave, with a capacity of 50 mL, followed by sealing and heating it at 180 °C for 12 h. After it gets cooled naturally to ambient temperature, the outcome was collected and washed through centrifugation with deionized water and ethanol (in equal ratio) several times to get the pH neutralized, followed by drying it in air at 60 °C for 12 h to acquire the rod-shaped α-Fe_2_O_3_ nanoparticles.

### 2.2. Fabrication of PDMS Film and PDMS/α-Fe_2_O_3_ Nanocomposite Film-Based Negative Tribo-Layer

**Figure 1:**
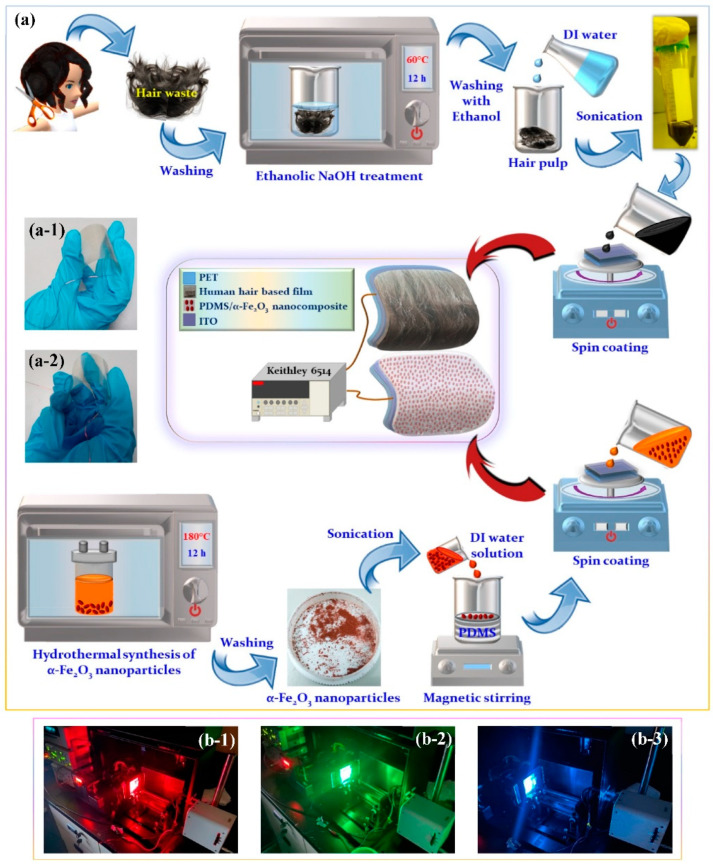
(**a**) Schematic demonstration of the fabrication procedure for triboelectric nanogenerator (TENG) consists of PDMS/α-Fe_2_O_3_ nanocomposite film (as the negative tribo-layer) and processed hair film (as the positive tribo-layer) along with the respective photographs of the (**a-1**) positive and (**a-2**) negative tribo-layers. (**b-1**–**b-3**) The photographs of the fabricated TENG measurement arrangement under illumination with different wavelengths.

**Figure 2:**
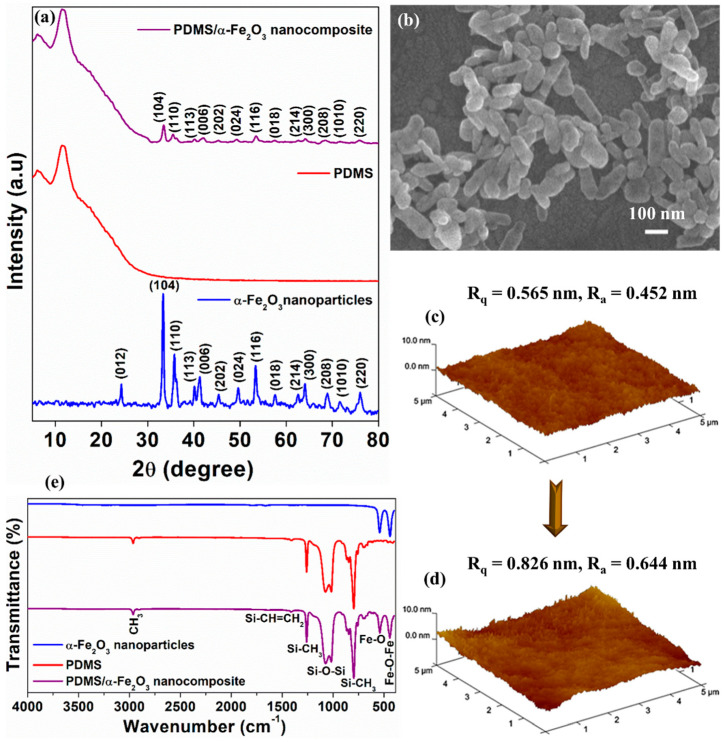
XRD patterns of (**a**) α−Fe_2_O_3_ nanoparticles, PDMS and PDMS/α−Fe_2_O_3_ nanocomposite. FESEM view of the (**b**) α−Fe_2_O_3_ nanoparticles. AFM analysis results of the (**c**) PDMS film and (**d**) PDMS/α−Fe_2_O_3_ nanocomposite film. (**e**) FTIR spectra of the α−Fe_2_O_3_ nanoparticles, PDMS and PDMS/α−Fe_2_O_3_ nanocomposite.

The PDMS source solution was developed by the blending process of Sylgard 184 elastomer (Dow Corning, Midland, MI, USA) and the curing agent (in 10:1 proportion) with addition of DI water through magnetic stirring. As shown in Figure 1a, the PDMS/α-Fe_2_O_3_ nanocomposite film (negative tribo-layer, Figure 1(a-2)) was produced over an Indium tin oxide (ITO)-coated flexible PET substrate with ITO as electrode material. First, the DI water solution of as-synthesized α-Fe_2_O_3_ nanoparticles (0.06 wt.% α-Fe_2_O_3_ nanoparticles) was mixed into PDMS (Sylgard 184 elastomer with curing agent in 10:1 ratio) by magnetic stirring for around 3–4 h till the solution becomes free from bubbles. The negative tribo-layers of the TENG were developed by spin-coating (at 1500 rpm for 60 s) the as-prepared solutions onto a 3 × 3 cm^2^ ITO-coated flexible PET which was cleaned up with the help of a N_2_ gun before spin coating, and shortly afterward the film was cured at 80 °C for 2 h, with further curing required at 105 °C for 2.5 h, to extract the existing DI water.

### 2.3. Fabrication of a Processed Human Hair-Based Positive Tribo-Layer

In the first step, 1.5 gm of waste hair (scalp hair waste samples of 20–30 year-old Asian women) was thoroughly cleaned with ethanol and DI water, and subsequently dried at 60 °C up to 30 min to immerse it in 40 mL of 1.5 M ethanolic NaOH (Shimakyu’s Pure Chemicals, Osaka, Japan) solution, followed by sealing and keeping it in an oven at 60 °C for 12 h. After getting this processed hair mass cooled naturally to room temperature, it was washed with ethanol through centrifugation and a homogeneous solution was prepared by adding an equal amount of DI water into it. As represented in Figure 1a, to fabricate the positive side (Figure 1(a-1)) of the TENGs the as-processed homogeneous solution was spin-coating (at 1500 rpm for 40 s) onto a 3 × 3 cm^2^ ITO-coated flexible PET substrate, which was cleaned by a N_2_ gun before the coating process started, followed by the drying process at 60 °C for 5 h after the spin-coating was done.

All the donors gave their informed consent for inclusion of the waste hair samples that they donated solely for the purpose of this research, before they participated in the study. The study was conducted in accordance with the Declaration of Helsinki, and the protocol was approved by the Human Test Ethics Committee of Chang Gung Medical Foundation (Application No.—2109220003, Date—24 September 2021).

### 2.4. Fabrication of the TENG Device

**Figure 3:**
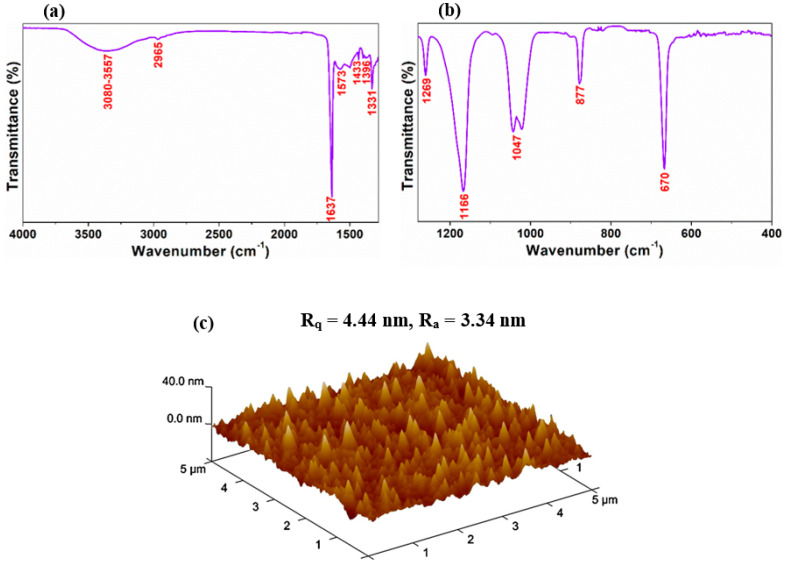
FTIR analysis result of the processed hair film over two certain regions (**a**) 4000−1280 cm^−1^ and (**b**) 1280−400 cm^−1^. (**c**) AFM analysis results of the processed hair film.

The self-powered photodetector based on the TENG device was composed of two parts, where the bottom part is a PDMS/α-Fe_2_O_3_ nanocomposite film-based negative tribo-layer with α-Fe_2_O_3_ nanoparticles working as effective light absorber and is constructed on a transparent and flexible ITO substrate and the upper part is the processed hair film-based positive tribo-layer fabricated on a transparent and flexible ITO substrate. Finally, conducting wires were connected to each of the ITO electrodes for electrical measurement. The as fabricated TENGs were operated in vertical contact-separation modes under dark and illumination conditions (Figure 1(b-1–b-3)).

### 2.5. Material Characterization and Electrical Measurement

The crystalline phase of the α-Fe_2_O_3_ nanoparticles and PDMS based composites were consistently studied by XRD (X-ray Diffraction for Thin Film Advanced Analysis and X-ray Powder Diffractometer equipped with a Cu Kα source, Rigaku_TTRAX Ⅲ, Tokyo, Japan). The surface morphologies and elemental analyses of all the as-synthesized samples were evaluated using FE-SEM integrated with energy-dispersive spectroscopy (EDS) (FE-SEM, JEOL JSM-7500F, Pleasanton, CA, USA) and AFM (Innova B067, Bruker Corp., Camarillo, CA, USA). The chemical bonding of all the samples were analyzed by Howard Infrared Spectrometer Microscope with ATR microscopy accessory (Germany Bruker, Vertex 80v, Ettlingen, Germany). The UV-vis spectra of α-Fe_2_O_3_ nanoparticles, PDMS and PDMS/α-Fe_2_O_3_ nanocomposite were analyzed by UV/Vis spectrophotometer (Jacso, V-650, Japan and Shimadzu UV-1900, Kyoto, Japan) recorded from 200 to 900 nm wavelength. All the electrical properties of as-fabricated TENG were evaluated by using a Keithley 6514 programmable electrometer and a low-noise current preamplifier (Stanford Research System Model SR570, Sunnyvale, CA, USA). Tunable LED light source (MIGHTEX, Uninanotech UNI-EGF-LED, Toronto, Ontario, Canada) was used to illuminate the device. The capacitances of the PDMS and PDMS/α-Fe_2_O_3_ nanocomposite films were scrutinized using an Agilent B1500A semiconductor device parameter analyzer (Santa Rosa, CA, USA).

## 3. Results and Discussion

### 3.1. Structural and Compositional Analysis

Figure 2a exhibits the XRD patterns of α-Fe_2_O_3_ nanoparticles, PDMS and the PDMS/α-Fe_2_O_3_ nanocomposite film to analyze the crystal structures of the as-prepared samples. The XRD patterns of α-Fe_2_O_3_ nanoparticles was consistent with the typical diffraction patterns (JCPDS card no. 33-0664) [25], and the non-existence of any further peak proved the emergence of the pure phase of hematite nanoparticles. As shown in the XRD pattern of PDMS/α-Fe_2_O_3_ nanocomposite film, the amorphous halo at 2θ = 12° was the indication of the unaffected crystalline domain of the PDMS films [26], while the crystallinity was also preserved due to the insertion of α-Fe_2_O_3_ nanoparticles. The XRD patterns affirmed the successful addition of pure α-Fe_2_O_3_ nanoparticles into the pure PDMS to prepare the PDMS/α-Fe_2_O_3_ nanocomposite with no alteration in the chemical composition. The FESEM image (Figure 2b) demonstrated the nanorod-like morphology of the as-synthesized α-Fe_2_O_3_ nanoparticles, with 50 nm widths and 150–200 nm lengths. The role of morphological changes of the electrification layer in determining the TENG output performance was examined using FE-SEM and AFM images of pure PDMS and PDMS/α-Fe_2_O_3_ nanocomposite surface (Figure S2 and Figure 2c,d). In contrast with the pure PDMS, the PDMS/α-Fe_2_O_3_ nanocomposite film features special surface morphology owing to the insertion of α-Fe_2_O_3_ nanoparticles into PDMS film. In the FESEM image of the PDMS/α-Fe_2_O_3_ nanocomposite film (Figure S2b), one can see the presence of well-defined α-Fe_2_O_3_ nanoparticles, evenly-distributed and properly embedded in PDMS matrix, therby improving the mechanical strength and surface roughness of the film [27]. The AFM analysis result demonstrates the root mean square (rms) roughness of the pure PDMS film to be 0.565 nm. Upon α-Fe_2_O_3_ nanoparticles insertion, the roughness was elevated up to 0.826 nm (Figure 2c,d) and this higher surface roughness beneficially enlarged the surface area.

The infrared transmittance spectra of functional groups were acquired from the pure PDMS, PDMS/α-Fe_2_O_3_ nanocomposite and α-Fe_2_O_3_ nanoparticles samples by using an FT-IR spectrometer accompanied with ATR microscopy accessory. As shown in Figure 2e the main FTIR spectrum of PDMS between the pristine PDMS and PDMS composite sample do not show considerable difference except the presence of characteristic peaks at about 540 cm^−1^ and 432 cm^−1^ matched with the bonds in α-Fe_2_O_3_ [28], that again indicates the successful formtion of PDMS/α-Fe_2_O_3_ nanocomposite. The typical peaks at wavenumbers of 2965 cm^−1^ (C-H methyl stretch), 1260 cm^−1^ (CH_3_ symmetric bending in silicon–methyl bond), identified the different existing functional groups in PDMS [29]. The wide polymer backbone absorption band found betwixt 1130 and 1000 cm^−1^ also characterises the PDMS [30]. The absorption band with lower intensity at 1410 cm^−1^ (for vinyl) and the absence of absorption band at 2140 cm^−1^ (for SiH), reveals the existence of unreacted vinyl groups in very small amount and no extra hydrosilane (SiH) groups [30]. Thus, FTIR analysis indicates that the insertion of α-Fe_2_O_3_ nanoparticles into pure PDMS to prepare the PDMS/α-Fe_2_O_3_ nanocomposite does not make any considerable transformation in the chemical bonds of PDMS essentially.

Figure S1 shows the energy-dispersive X-ray spectroscopy (EDS) analysis results for the pure PDMS, pure α-Fe_2_O_3_ nanoparticles and PDMS/α-Fe_2_O_3_ nanocomposite. Both Fe and O are found in almost expected amount due to the integration of the α-Fe_2_O_3_ nanoparticles into the pure PDMS, to form the PDMS/α-Fe_2_O_3_ nanocomposite-based negative tribo-layer.

IR spectroscopy for the processed hair film had been used to characterize the compounds and to recognize the purity of a material, where infrared radiation with a clear span of 4000 to 400 cm^−1^ was used to scan the sample. As represented in Figure 3a,b a broad peak arised at around 3080–3557 cm^−1^, possibly arose from the asymmetric and symmetric stretching modes of H−O−H and the stretching of N−H group [31]. The respective expansion of this peak for processed hair film in comparison with that of the untreated hair could be caused by an additional modification of its chemical structure due to the heat treatment at 60 °C [32]. The small peak at 2965 cm^−1^ originated from the asymmetric stretching mode of CH_3_. The three important amide bands attributed to the peptide bonds in untreated hair deveopes at 1700−1580 cm^−1^ (amide I); 1580−1500 cm^−1^ (amide II); and 1320−1210 cm^−1^ (amide III) [33]. Although the weak and the very strong absorption peaks detected at 1573 cm^−1^ and 1637 cm^−1^, respectively, are not generated from the pure amide modes, yet preferably from amides that hydrolyzed into carboxylic acid groups after the reaction with strong bases, while the peak at around 1637 cm^−1^ probably resulted from the overlapping of N−H bending and C=C stretching modes [31]. The very weak peaks emerged at 1573 cm^−1^ and 1396 cm^−1^ possibly originated from the symmetric and antisymmetric stretching modes of −COO−, developed due to ionization of the carboxylic acid groups in the ethanolic NaOH. The weak peak appeared at 1433 cm^−1^ arose due to the vibration of the CH_2_ group in scissoring mode. The strong peak found at 1331 cm^−1^ possibly originated from the S=O groups emerged by the oxidation of cysteine disulfide cross-links, exists in the untreated human hair [31]. The peak at approximately 1269 cm^−1^ corresponds to the N-H bending mode, while 1166 cm^−1^ represented the ester C–O asymmetric stretching mode [34]. The intense absorption at 1047 cm^−1^ was may be because of the appearance of the C−O stretching in the alcohol residue still existing in the processed hair film also because of the cysteic acid [31]. The peak at 877 cm^−1^ arose from the distortion of the hydrocarbons present in the keratin proteins, and the sharp peak at 670 cm^−1^ was designated to the C−OH out-of-plane bending mode [31]. Thus the FTIR study devulged some deformation in the chemical structure of natural untreated human hair due to the NaOH treatment at 60 °C.

As shown in Figure S3a, the EDS analysis revealed the weight and atomic percentages of vital chemical elements present in processed hair film were similar to the regular amounts traced in natural untreated human hair [31].

The FE-SEM and AFM image in Figure S3b and Figure 3c represents the surface morphology of the processed hair film. The as-prepared film evinces a nanoisland like surface morphology without any cracks or trace of undissolved hair with uniform coverage over the ITO electrode. The AFM analysis offered more insight into the surface roughness, and three-dimensional features along with the surface morphology of the as-prepared processed hair film, revealing that it is a compact film with the root mean square (rms) surface roughness of 4.44 nm (Figure 3c).

### 3.2. Electrical Performance

**Figure 4:**
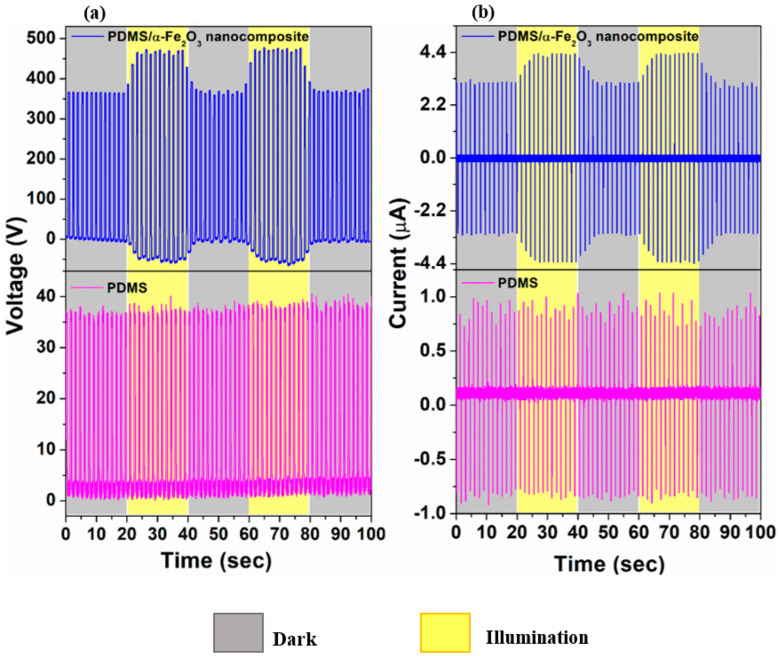
(**a**) Voltage and (**b**) current outputs of PDMS and PDMS/α−Fe_2_O_3_ nanocomposite−based TENGs in the dark and under illumination.

As shown in Figure 4, the performance of the as fabricated TENGs, operated in vertical contact-separation mode was signalized by measuring its output voltage and current under various light induced conditions. In dark state, the voltage and current signals are solely from the mechanical energy provided by the linear stepper motor during the periodic contact and separation cycle of the TENG device. As the α-Fe_2_O_3_ nanoparticles were introduced into the PDMS to form the PDMS/α-Fe_2_O_3_ nanocomposite, the magnitude of mean peak-to-peak output voltage was intensified from 37 to 367 V with the corresponding output current showing similar way of variation from 1.74 μA to 6.41 μA. This enhancement in output signals may be due to the combined effect of special surface morphology of the film with elevated surface roughness, effective contact area and the enriched charge trapping occurring from the insertion of high dielectric constant α-Fe_2_O_3_ nanoparticles into the pure PDMS film, which assists to enhance charge transfer process during the triboelectrification mechanism, as explained thoroughly in our previous study, Reference [20]. Figure 4a,b describes the change in voltage and current outputs of the TENGs during the switching on and off the white light (intensity—13.39 mW cm^−2^) repeatedly. The voltage and current amplitude (peak-to-peak value) was immediately intensified up to approximately 1.4 times, after the PDMS/α-Fe_2_O_3_ nanocomposite based-TENG was exposed to the illumination and once the illumination was cut off, the voltage and current amplitudes recover quickly to the original value, although the bare PDMS based-TENG did not exhibit any further change in the output signals. These cycles of the periodic illumination indicate outstanding repeatability of the photo-enhancement.

Since the electric signal produced by the PDMS/α-Fe_2_O_3_ nanocomposite-based TENG is dynamic, it could be puzzling to calculate an accurate value of the response time. Nevertheless, it can be fairly evaluated. As shown in Figure 4a, the voltage amplitude has a considerable step increment in the first cycle just after turning on the light and step reduction for turning off the light, indicating an immediate response to the illumination. This first step up and step down variation taking place within 150 ms. Consequently, the response time of this photo-induced TENG is less than 150 ms, which is supercilious to numerous recently reported self-powered photodetectors [18].

**Figure 5:**
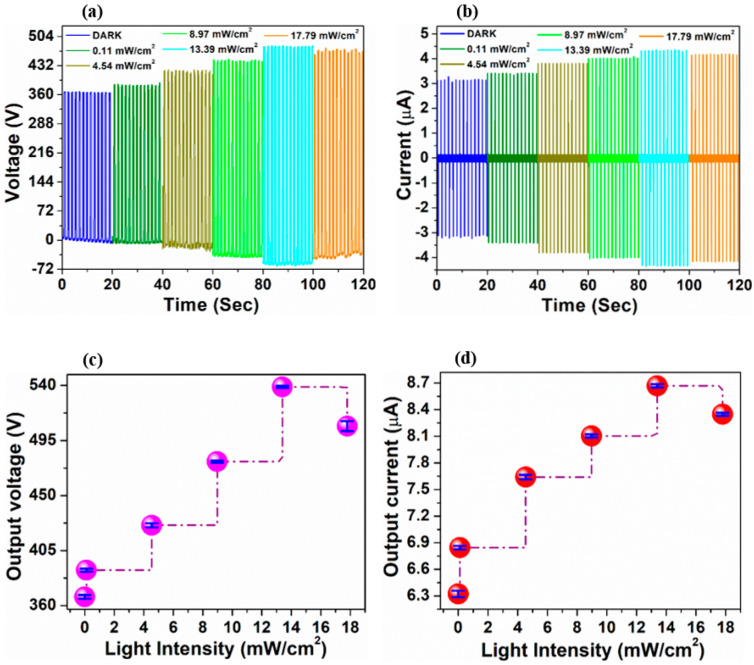
Variation in (**a**) voltage and (**b**) current signal of PDMS/α−Fe_2_O_3_ nanocomposite−based TENG upon illumination with increasing light intensity. The variation of the mean magnitudes of peak−to−peak (**c**) voltage and (**d**) current outputs (with standard deviations) of the TENG under illumination (white light) with different intensities.

It was observed that the amplitude of the output voltage and current signals from the PDMS/α-Fe_2_O_3_ nanocomposite-based TENG under illumination intensifies as the applied light intensity rises from 0.11 to 13.39 mW cm^−2^ (Figure 5). However, it should be mentioned that the output signals suffered from a very tiny downswing when the intensity of light is over 13.39 mW cm^−2^ that is 17.79 mW cm^−2^, this is likely attributed to a decay of PDMS/α-Fe_2_O_3_ nanocomposite film owing to the high intensity light and a longtime measurement. This light intensity dependency of the output signals qualifies the TENG to be a realizable perspective for photo-detection.

Moreover, the responsivity (*R*) can be interpreted using the following equation,

(1) $$R=\frac{\Delta V}{A\Delta P}$$

where Δ*V* is the change in the amplitude of output voltage arising from the increase in light intensity, *A* is the sample area under illumination, and Δ*P* is the intensity of light [35]. The responsivity can be calculated by Equation (1), which originates a value of 1.42 V mW^−1^, presenting a remarkable improvement over the recent TENG-based self-powered photodetectors.

**Figure 6:**
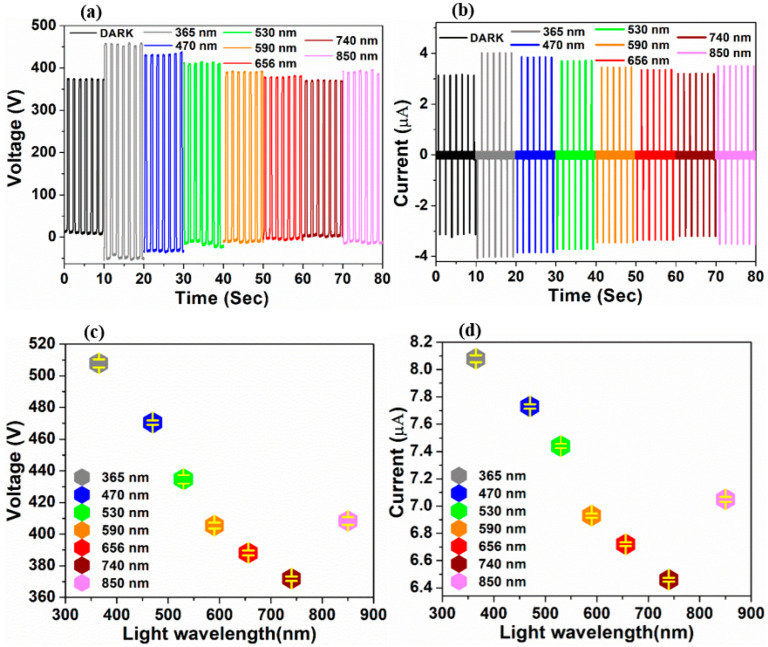
Variation in (**a**) voltage and (**b**) current signal of PDMS/α−Fe_2_O_3_ nanocomposite−based TENG upon illumination with several different wavelengths. The variation of the mean magnitudes of peak−to−peak (**c**) voltage and (**d**) current outputs (with standard deviations) of the TENG under illumination with different wavelengths.

Further, the response of output signal from the as fabricated PDMS/α-Fe_2_O_3_ nanocomposite-based TENG, on different wavelengths of lights was analyzed, as the ultra broad-band detection capability is one another major quality for an acceptable photodetector. As depicted in Figure 6, the output signals manifest signification changes in their magnitudes under illumination (intensity-13.39 mW cm^−2^) over the near UV, visible and near IR regions, which specify an ultra broad-band detection capability. The pattern of variation of the output signal under illumination of different wavelengths (Figure 6c,d) conforms to the UV–Vis absorption of α-Fe_2_O_3_ nanoparticles [36].

### 3.3. Mechanism Discussion

To interpret the triboelectric charge-transfer mechanism for the photo induced enhancement of the PDMS/α-Fe_2_O_3_ nanocomposite-based TENG, the contact-separation process of TENG is split into two parts for detailed discussion.

As represented in Figure S4a–e, without illumination, the TENG can simply convert vibrating energy into electrical energy under repeated external force, applied through the mover of linear stepper motor. In the initial stage, no charge occurs on the friction surface of the PDMS/α-Fe_2_O_3_ nanocomposite film and processed hair film. Following the two friction layers come into physical contact, negative tribo-charges are assembled on the surface of PDMS/α-Fe_2_O_3_ nanocomposite film, developing identical amount of tribo-positive charges on the processed hair film surface because of the difference in electron affinity. After that, as the TENG device started getting separated under the external applied force, the tribo-charges will build a time-varying electric field in space, as well will induce the positive and negative charges on the corresponding ITO electrodes respectively. In the course of this process, these inductive electrons will flow across the external circuit to balance the generated potential difference and attain electrostatic equilibrium eventually. Conversely, when these two frictional layers come back to the initial state from the top displaced position, the electrons will move at reversed direction till the complete contact, in this way generated an oscillating electric output signal.

**Figure 7:**
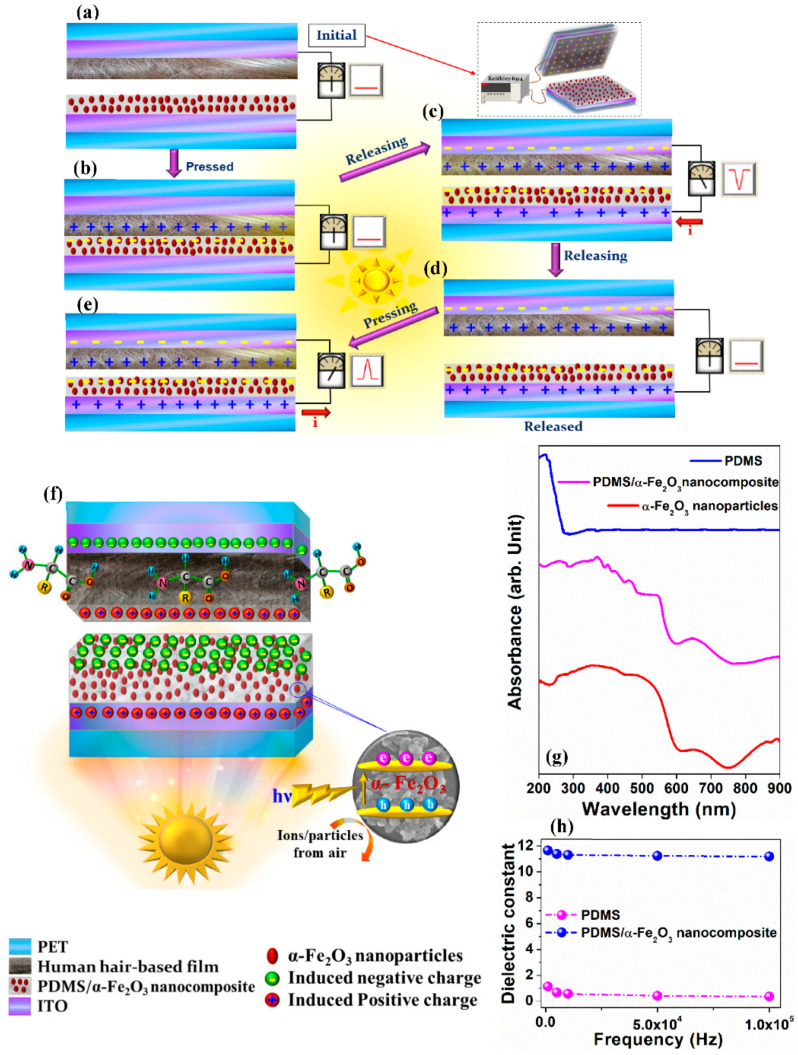
(**a**–**e**) The working mechanism of as fabricated TENG under illumination. (**f**) Schematic of the triboelectric charge transfer procedure in the TENG consists of the PDMS/α-Fe_2_O_3_ nanocomposite and processed hair film friction layers under illumination. (**g**) UV-vis absorption spectrum of the α-Fe_2_O_3_ nanoparticles, PDMS and PDMS/α-Fe_2_O_3_ nanocomposite. (**h**) Dielectric constant of the PDMS and PDMS/α-Fe_2_O_3_ nanocomposite films as a function of frequency.

Under illumination condition, this TENG executes a double conversion mechanism as shown in Figure 7a–f. Other than the benefaction from the triboelectricity, photoelectric conversion is one other source for this improvement in electric output. Under illumination condition also, following the conventional mechanism of triboelectric conversion by the coupling of contact electrification and electrostatic induction, once the electrification layers come into contact with each other, due to huge differences in the electron affinities between the PDMS/α-Fe_2_O_3_ nanocomposite and human hair, surface charge transfer occurs, resulting in the development of opposite charges over the surface of those friction layers (Figure 7b). At this stage, there is no electric potential difference generated between the two back electrodes as with opposite charges coexist at the same plane [37]. Upon releasing the two parts of the device, a potential difference is then developed betwixt the two ITO electrodes as the charge accumulated on the contact induces opposite charges on the back electrodes (ITO electrodes), generating current flowing through the external circuit from the upper part to the lower part of the TENG device (Figure 7c). Immediately after the application of illumination, a lot of electron-hole pairs are produced within the PDMS/α-Fe_2_O_3_ nanocomposite layer, resulted from photon absorption, owing to the superior absorption characteristics of the α-Fe_2_O_3_ nanoparticles. As shown in the Figure 7g, after the insertion of α-Fe_2_O_3_ nanoparticles into the PDMS matrix, distinct peaks appeared due to the α-Fe_2_O_3_ nanoparticles absorption characteristics over the near UV, visible and near IR regions, while the bare PDMS exhibited no considerable absorption peaks except an absorption peak at approximately 220 nm in the middle-UV region [38]. As demonstrated in Figure 7f, these photo generated electrons get quickly captured by the α-Fe_2_O_3_ nanoparticles, embedded in the negative tribo-layer, that is recognized for its surpassing charge trapping property [39], confirmed by the appreciable enhancement in the dielectric properties of the PDMS based-composite owing to the insertion of α-Fe_2_O_3_ nanoparticles into the pure PDMS (Figure 7h), while the generated holes then tend to get neutralized by the recombination with adsorbing negatively charged particles or ions from air [40]. Considering comparatively lower dielectric constant of the as prepared PDMS film (Figure 7h), as the general distinction of dielectric property is noticed because of the changes of space charge/interfacial polarization developing from differences in the surface to volume ratio of the presented structure morphology [20,41,42,43]. Hence, these trapped electrons successively, are amplified to the previously-available negative tribo-charges, resulting in an enhancement in the surface charge density of PDMS/α-Fe_2_O_3_ nanocomposite film, thereby amplifying the output performance of this TENGs upon light illumination. Afterwards, the two tribo-layers are fully released (Figure 7d), the output signals fall to zero. When the TENG is pressed again (Figure 7e), electrons are pushed back from the top to the bottom current collector, since the electrostatic potential difference is recompensed for by the opposite charges on both tribo-layers, till the induced charges get neutralized.

Therefore, it has been observed that the as-fabricated TENG exhibits fast response on both white light and monochromatic light with appreciable differences in the output signal responses under different intensities and wavelengths of light covering from the near UV to near IR regions, hence providing a novel methodology towards self-powered photodetection.

The processed hair film based positive tribo-layer manifests the TENG output performance considerably alike to that of the untreated natural hair waste, as explained minutely in our previous study, Reference [20]. Therefore, it is anticipated that the as prepared PDMS/α-Fe_2_O_3_ nanocomposite film will be capable of harvesting human hair movement reliably, in consequence of spontaneous gesture, in that way meeting users’ different functional necessity and executing the aforesaid broadband photodetection during any activities under the sun with this PDMS/α-Fe_2_O_3_ nanocomposite film fixed inside some transparent hair accessories, for example a hair clip, hairband, hair cap and head cap, even also helmet.

## 4. Conclusions

To sum up, the PDMS/α-Fe_2_O_3_ nanocomposite and processed hair film-based TENG with both superlative photoelectric and triboelectric properties has been exhibited, which illustrates an extraordinary performance without any dependence on an external power source. This well-made bio-TENG based on the coupling of triboelectric and photoelectric conversion mechanisms achieved superior photo-induced enhancement with the output voltage and current were increased by 46.73% and 37.1%, respectively. What is more, this TENG exhibits a high photo responsivity (1.42 V mW^−1^) and rapid response (<150 ms), on both white light and monochromatic light covering from the near UV to near IR regions. By scrutinizing the mechanism, we discovered that the major reason behind this TENG output boosting upon light illumination is the generation of a large number of photoelectrons trapped on the surface of negative electrification layer due to the combined effect of superior absorption characteristics and surpassing charge trapping property of the α-Fe_2_O_3_ nanoparticles, ensuing in an amplification in surface charge density. Additionally, the use of the processed human hair waste-based film as the positive tribo-layer, mitigates the challenge to waste management systems by extending the human hair waste utilization while securing the social and environmental welfare. This work manifests that the flexible, bio-based TENG is a very probable choice for the applications in self-powered and ultra-broadband light detection while generating significant socioeconomic benefits for people, by developing a promising approach towards the complete utilization systems for human hair waste.

## Data Availability

Data are contained within the article and Supplementary Material.

## Supplementary Materials

The following are available online at https://www.mdpi.com/article/10.3390/nano12071147/s1, Figure S1: EDS analysis results of the (a) PDMS film (b) α-Fe_2_O_3_ nanoparticles and (c) PDMS/α-Fe_2_O_3_ nanocomposite film, Figure S2: FESEM images of the (a) PDMS film and (b) PDMS/α-Fe_2_O_3_ nanocomposite film, Figure S3: (a) Results of the EDS analysis for processed hair film. (b) FESEM image of the processed hair film, Figure S4: Working mechanism of the TENG consist of PDMS/α-Fe_2_O_3_ nanocomposite film (as the negative tribo-layer) and human hair (as the positive tribo-layer) under dark state: (a) Initial stage of operation of the TENG (b) The first contact state without any output signal arises (c) On releasing state, a negative electrical output signal arose (d) After the friction layers get completely released, the output signal drops to zero (e) On subsequent pressing, a positive electrical signal is produced.
