# Supplementary material for: α-Fe2O3 Nanoparticles Aided-Dual Conversion for Self-Powered Bio-Based Photodetector
Source: Nanomaterials (Basel). 2022 Mar 30;12(7):1147. doi: 10.3390/nano12071147 (PMC9000849; doi:10.3390/nano12071147)
Supplement: Supplementary file 1 [file nanomaterials-12-01147-s001.zip › nanomaterials-1632216-supplementary.pdf]

Supplementary Materials

α-Fe_2O_3 Nanoparticles Aided-Dual Conversion for Self-Powered Bio-based Photodetector

Ishita Chakraborty [1], Sz-Nian Lai [2,3], Jyh-Ming Wu [2,4] and Chao-Sung Lai [1,5,6,7,*]

[1] Department of Electronic Engineering, Chang Gung University, Guishan District, Taoyuan City 33302, Taiwan; d0627107@cgu.edu.tw
[2] Department of Materials Science and Engineering, National Tsing Hua University, Hsinchu 30010, Taiwan; snlai712@gapp.nthu.edu.tw (S.-N.L.); jmwuyun@gapp.nthu.edu.tw (J.-M.W.)
[3] Ph.D. Program in Prospective Functional Materials Industry, National Tsing Hua University, Hsinchu 30010, Taiwan
[4] High Entropy Materials Center, National Tsing Hua University, Hsinchu 30010, Taiwan
[5] Artificial Intelligence and Green Technology Research Center, Chang Gung University, Guishan District, Taoyuan City 33302, Taiwan
[6] Department of Materials Engineering, Ming Chi University of Technology, Taishan District, New Taipei City 24301, Taiwan
[7] Department of Nephrology, Chang Gung Memorial Hospital, Guishan District, Taoyuan City 33305, Taiwan
* Correspondence: cslai@mail.cgu.edu.tw

Citation: Chakraborty, I.; Lai, S.-N.; Wu, J.-M.; Lai, C.-S. α-Fe_2O_3 Nanoparticles Aided-Dual Conversion for Self-Powered Bio-based Photodetector. *Nanomaterials* **2022**, *12*, 1147. https://doi.org/10.3390/nano12071147

Academic Editor: Werner Blau

Received: 24 February 2022
Accepted: 27 March 2022
Published: date

Publisher's Note: MDPI stays neutral with regard to jurisdictional claims in published maps and institutional affiliations.

Copyright: © 2022 by the authors. Submitted for possible open access publication under the terms and conditions of the Creative Commons Attribution (CC BY) license (https://creativecommons.org/licenses/by/4.0/).

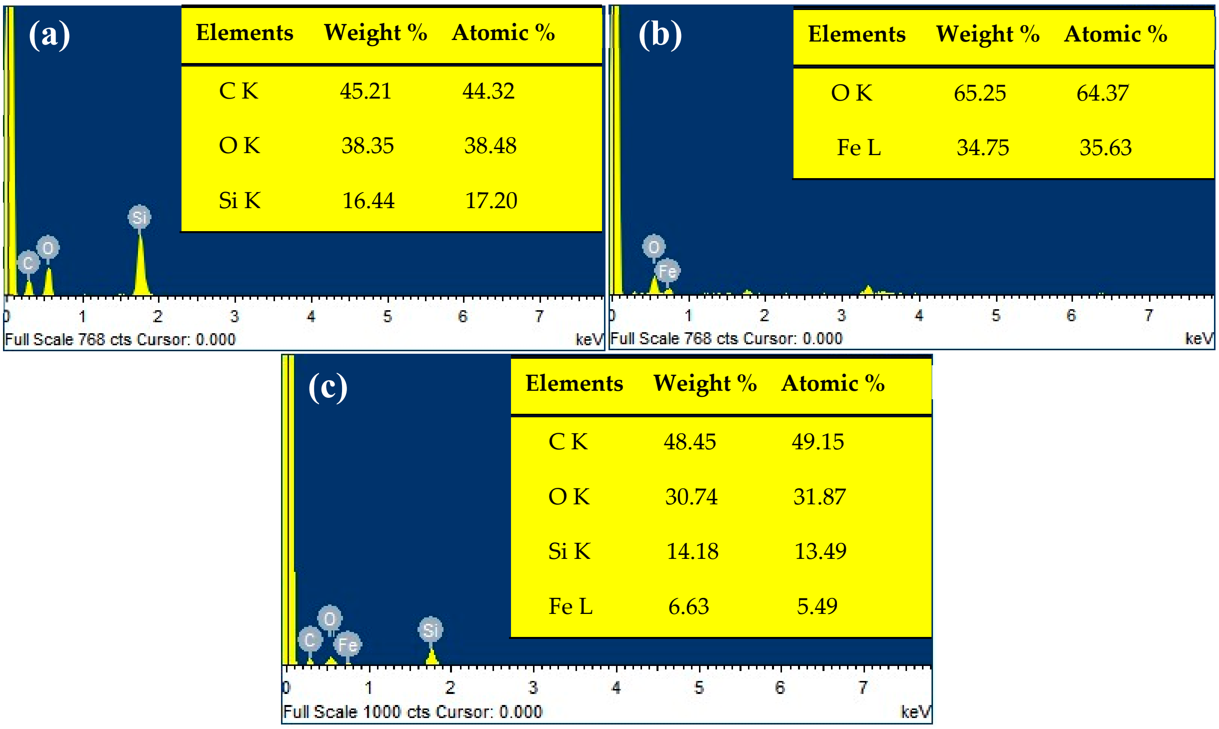

(a)

Elements	Weight %	Atomic %
C K	45.21	44.32
O K	38.35	38.48
Si K	16.44	17.20

(b)

Elements	Weight %	Atomic %
O K	65.25	64.37
Fe L	34.75	35.63

(c)

Elements	Weight %	Atomic %
C K	48.45	49.15
O K	30.74	31.87
Si K	14.18	13.49
Fe L	6.63	5.49

Figure S1. EDS analysis results of the (a) PDMS film (b) α-Fe_2O_3 nanoparticles and (c) PDMS/α-Fe_2O_3 nanocomposite film.

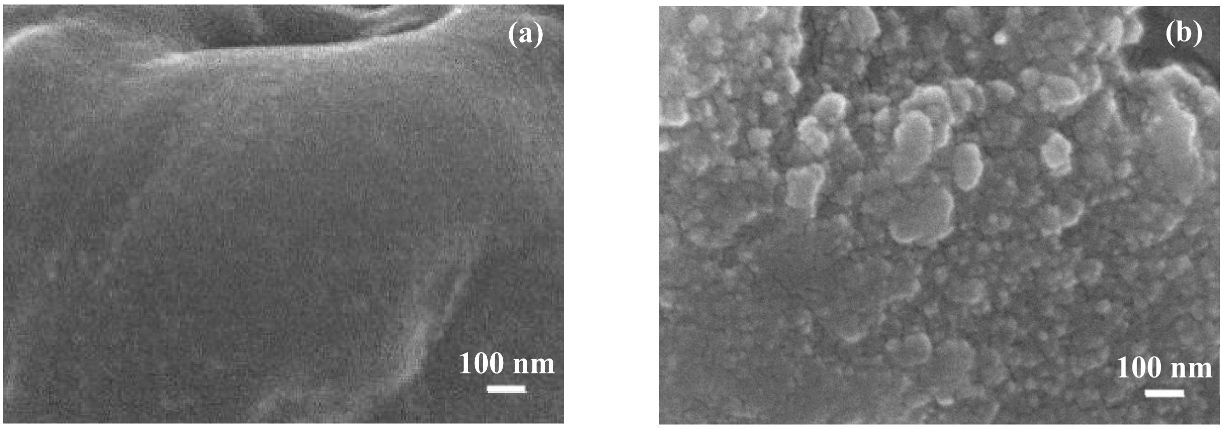

Figure S2. FESEM images of the (a) PDMS film and (b) PDMS/α-Fe_2O_3 nanocomposite film.

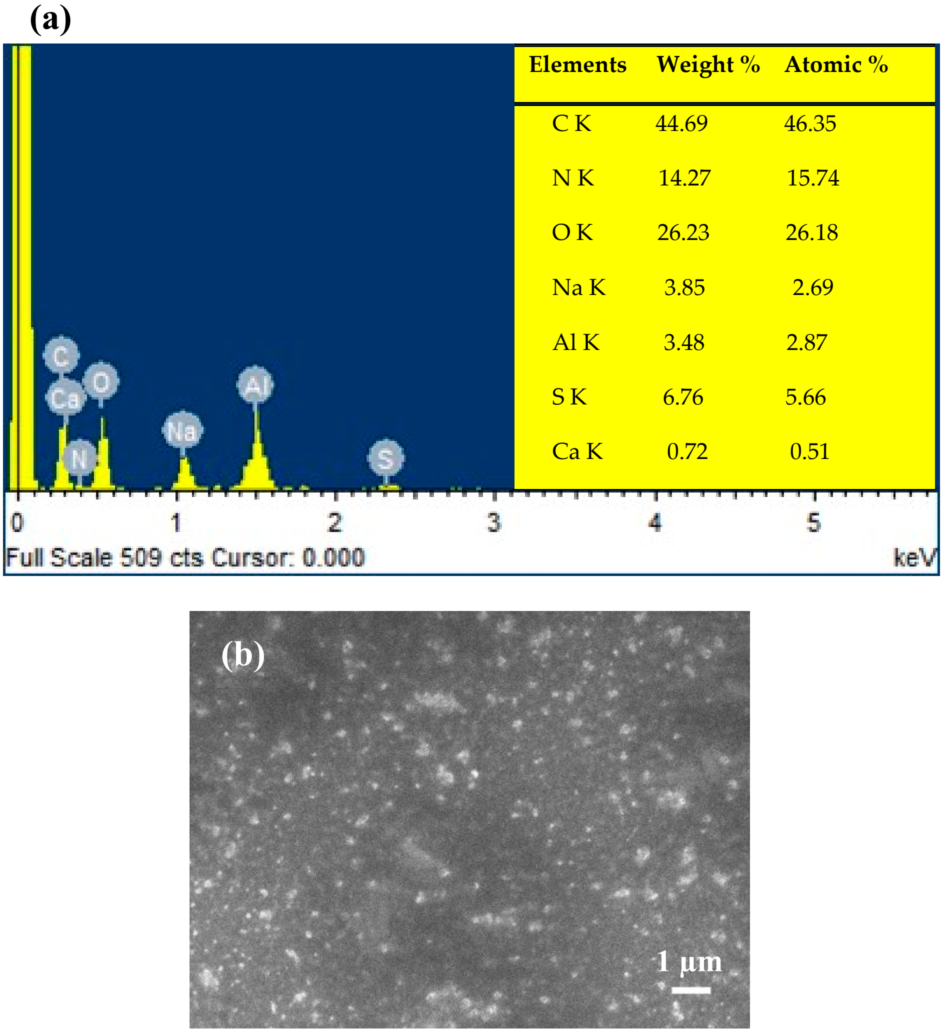

Elements	Weight %	Atomic %
C K	44.69	46.35
N K	14.27	15.74
O K	26.23	26.18
Na K	3.85	2.69
Al K	3.48	2.87
S K	6.76	5.66
Ca K	0.72	0.51

Figure S3. (a) Results of the EDS analysis for processed hair film. (b) FESEM image of the processed hair film.

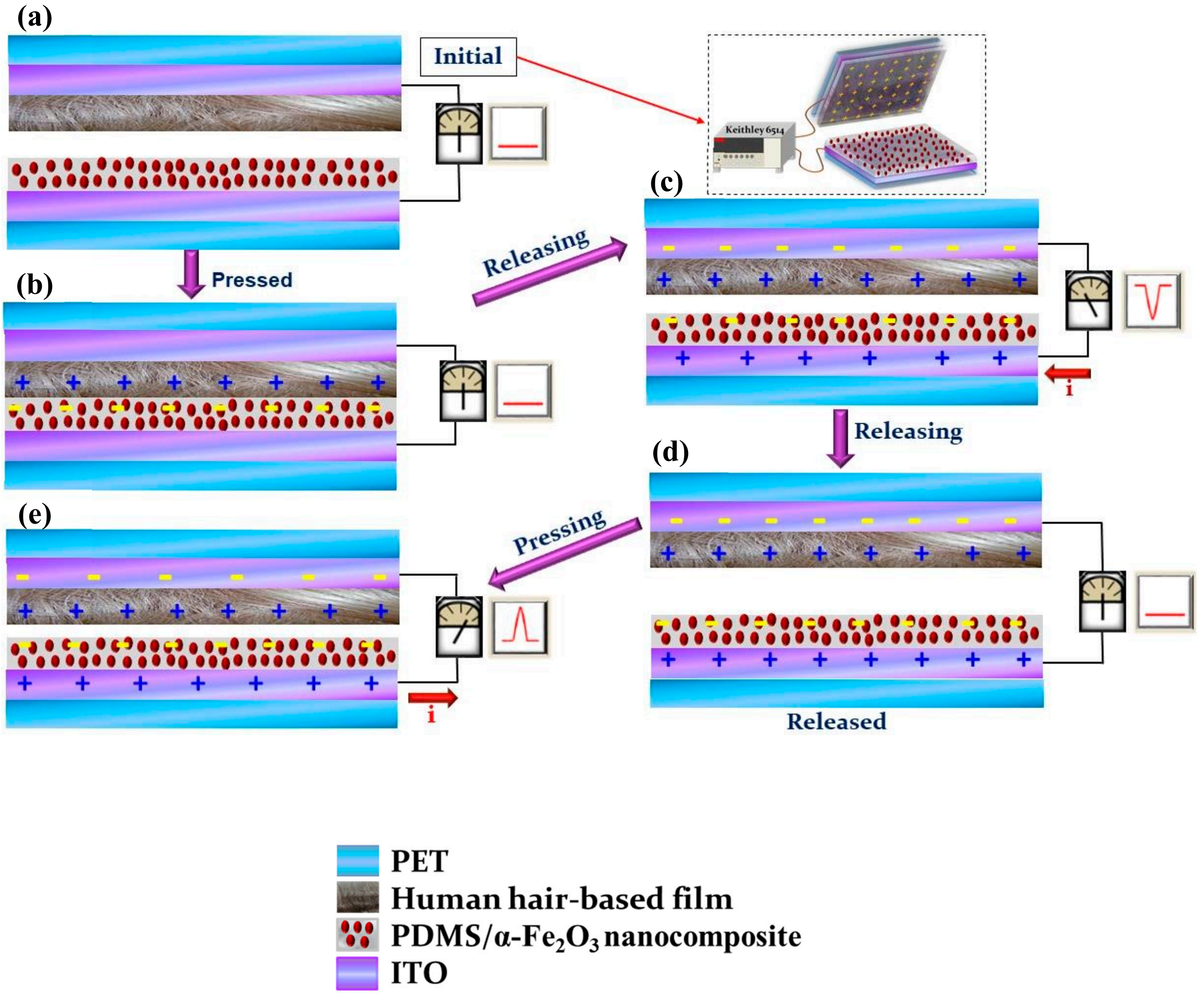

Figure S4. Working mechanism of the TENG consist of PDMS/α-Fe_2O_3 nanocomposite film (as the negative tribo-layer) and human hair (as the positive tribo-layer) under dark state: (a) Initial stage of operation of the TENG (b) The first contact state without any output signal arises (c) On releasing state, a negative electrical output signal arose (d) After the friction layers get completely released, the output signal drops to zero (e) On subsequent pressing, a positive electrical signal is produced.
